# Inhibitory Activity of Plant Essential Oils against *E. coli* 1-Deoxy-d-xylulose-5-phosphate reductoisomerase

**DOI:** 10.3390/molecules24142518

**Published:** 2019-07-10

**Authors:** Ge Yan, Bo-Rong Zhu, Fang-Lin Tian, Xian Hui, Heng Li, Yi-Ming Li, Wen-Yun Gao

**Affiliations:** 1 National Engineering Research Center for Miniaturized Detection Systems and College of Life Sciences, Northwest University, 229 North Taibai Road, Xi’an 710069, China; 2 School of Pharmacy, Shanghai University of Traditional Chinese Medicine, 1200 Cailun Road, Shanghai 201203, China

**Keywords:** plant essential oils, antibacterial mechanism, 1-deoxy-d-xylulose-5-phosphate reductoisomerase, inhibition

## Abstract

The rate-limiting enzyme of the 2-methyl-d-erythritol-4-phosphate (MEP) terpenoid biosynthetic pathway, 1-deoxy-d-xylulose-5-phosphate reductoisomerase (DXR), provides the perfect target for screening new antibacterial substances. In this study, we tested the DXR inhibitory effect of 35 plant essential oils (EOs), which have long been recognized for their antimicrobial properties. The results show that the EOs of *Zanbthoxylum bungeanum* (ZB), *Schizonepetae tenuifoliae* (ST), *Thymus quinquecostatus* (TQ), *Origanum vulgare* (OV), and *Eugenia caryophyllata* (EC) displayed weak to medium inhibitory activity against DXR, with IC_50_ values of 78 μg/mL, 65 μg/mL, 59 μg/mL, 48 μg/mL, and 37 μg/mL, respectively. GC-MS analyses of the above oils and further DXR inhibitory activity tests of their major components revealed that eugenol (EC) and carvacrol (TQ and OV) possess medium inhibition against the protein (68.3% and 55.6%, respectively, at a concentration of 20 μg/mL), whereas thymol (ST, TQ, and OV), carveol (ZB), and linalool (ZB, ST, and OV) only exhibited weak inhibition against DXR, at 20 μg/mL (23%−26%). The results add more details to the antimicrobial mechanisms of plant EOs, which could be very helpful in the direction of the reasonable use of EOs in the food industry and in the control of phytopathogenic microbials.

## 1. Introduction

It has long been recognized that plant essential oils (EOs) exhibit many types of bioactivities, such as antimicrobial, antiviral, antimycotic, anti-toxigenic, antiparasitic, and insecticidal properties [1], and their microbicidal effects have been well documented [2]. Recent studies in this area have focused largely on the determination of the antimicrobial activities of various EOs against different microbials, such as human pathogenic bacteria [3,4], phytopathogenic bacteria [5,6], foodborne pathogens [7,8], food-spoilage yeasts [9], phytopathogenic fungi [10] and their potential applications. Meanwhile, some investigations have focused on the mechanisms of the bactericidal actions of EOs [11,12,13,14,15,16]. 

**Figure 1:**
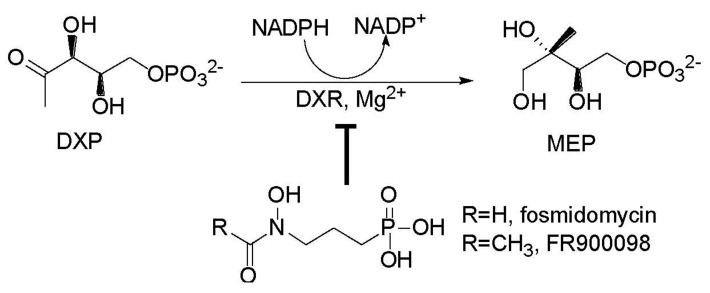
The first committed step of the 2-methyl-d-erythritol-4-phosphate (MEP) terpenoid biosynthetic pathway and its inhibitors.

Emerging drug resistance compels additional research in developing novel antibiotics exhibiting distinguished mechanisms; enzymes involved in the 2-methyl-d-erythritol-4-phosphate (MEP) terpenoid biosynthetic pathway are attractive targets since this newly established pathway exists only in human pathogens and not in human beings, and disruption of the pathway is lethal for the bacteria [17]. The speed-limiting enzyme 1-deoxy-d-xylulose-5-phosphate reductoisomerase (DXR) in the MEP pathway has been acknowledged as a superior target for screening antibiotics. It catalyzes the conversion of 1-deoxy-d-xylulose-5-phosphate (DXP) to MEP in the presence of a divalent cation and Nicotinamide Adenine Dinucleotide Phosphate Reduced (NADPH) through a retroaldol-aldol mechanism (see Figure 1) [18,19,20]. A recent study showed that fosmidomycin that was previously isolated from *Streptomyces lavendulae* and its analog FR900098 are not only two potent DXR inhibitors but also possess strong antimicrobial activity [21]. Clinical trials have pointed out that these two compounds are effective in treating *Plasmodium falciparum*, the parasite responsible for malaria [22]. Hundreds of structural analogues of fosmidomycin and FR900098 have been prepared and their activities against DXR have been evaluated [23,24]. Kaiser and co-workers tested Mediterranean plants and found that the leaf extracts of *Cercis siliquastrum* strongly inhibited DXR, but no specific compound was elucidated [25]. In our search for DXR inhibitors in natural sources, we found that gallocatechin gallate and theaflavin 3,3′-digallate, which were isolated from tea, are potent DXR inhibitors [26,27,28]. Moreover, we also determined that eugenol and carvacrol, which were obtained from plant essential oils, possess medium suppressive activity against DXR [29].

In this study, the *E. coli* DXR inhibitory activity of 35 plant EOs was evaluated and the results show that the oils from *Zanbthoxylum bungeanum* (ZB), *Schizonepetae tenuifoliae* (ST), *Thymus quinquecostatus* (TQ), *Origanum vulgare* (OV) and *Eugenia caryophyllata* (EC) exhibit weak to medium inhibition against DXR at a concentration of about 0.05 mg/mL. GC-MS analyses of the five oils revealed their major components, whose DXR inhibitory activities were further assayed. Here, we disclose all the experimental details.

## 2. Results

### 2.1. Isolation of Plant EOs

To obtain enough EO from each plant, we took different amounts of plant materials for the hydrodistillation. The yields of the EOs of the selected plants are listed Table S1 in the supporting information (SI). The EO yield of the dry flower buds of EC is about 10%, which represents the highest oil content among all the plants involved in this study. Although the dry roots of both AD and CLo were utilized to extract the EOs, CLo gave a yield of more than 6%, the second highest oil content, whilst AD produced the lowest yield among all the plants investigated. Unexpectedly, the oil yields of AA and AAr were almost at the same level as that of AD, although the dry aerial parts of AA and AAr were hydrodistilled. The possible reason could be that the EOs in these two plants mainly comprise low boiling point components that are lost during air-dry. The EO yields of the other plants were at normal levels.

### 2.2. Photometric Assay of the DXR Inhibitory Activity of Plant EOs

**Figure 2:**
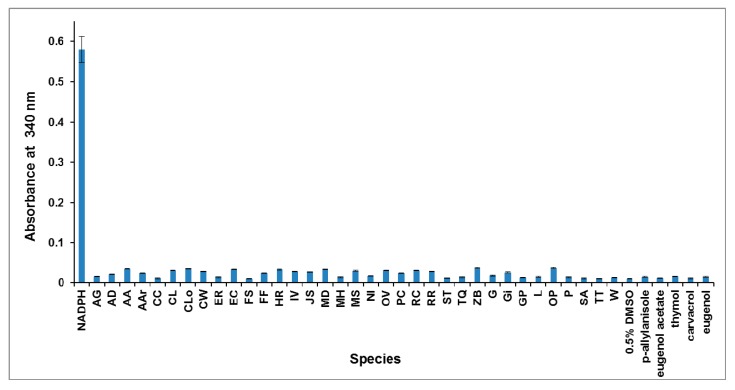
The UV absorbance of NADPH, plant essential oils (Eos), individual EO components, and DMSO at 340 nm. NADPH (0.15 mM in 100 mM Tris-HCl, pH 7.4); Plant EOs and individual EO component at 50 μg/mL in methanol; DMSO at 0.5% in water.

Plant EOs normally contain aromatic components that may have an absorbance at 340 nm, and may interfere with the photometric determination when added to the assay mixture. Therefore, we tested the absorbance of all 35 EOs and the individual EO compound containing an aromatic ring (p-allylanisole, eugenol acetate, thymol, carvacrol, and eugenol) at 50 μg/mL and 340 nm, respectively. The results, depicted in Figure 2, show that the A_340_ of each EO and EO compound used in this study was less than 0.03 at this concentration. Therefore, the maximum concentrations of the EOs used in the assays were controlled under 0.1 mg/mL to avoid possible interference. Meanwhile, we also measured the A_340_ of DMSO at a concentration of 0.5% (*V*/*V*) because 0.5% DMSO is enough to solubilize the EOs and the individual EO compound in aqueous medium. The results indicate that a DMSO concentration of 0.5% (*V*/*V*) was acceptable for the screening procedure (Figure 2), so we chose 0.5% DMSO in the bioassay. 

**Figure 3:**
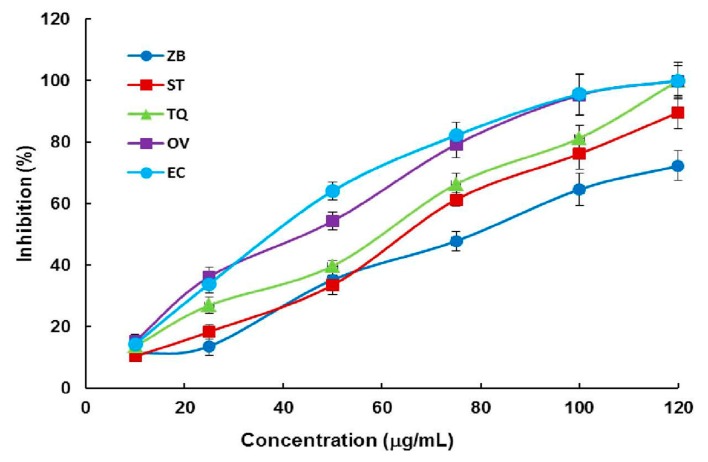
Concentration-dependent 1-deoxy-d-xylulose-5-phosphate reductoisomerase (DXR) inhibition manners of the EOs of ZB, ST, TQ, OV, and EC.

Using the photometric assay method, we first checked the influence of DMSO on the activity of DXR at a concentration of 0.5% and the results indicate that it did not retard the reaction at this concentration. So, 0.5% DMSO was used for the following test. Then, we evaluated the DXR inhibitory activity of all 35 plant EOs at final concentrations of around 0.05 mg/mL and the results (Table S1 in SI) show that at the selected concentration, a large part of the EOs exhibited very weak or even undetectable DXR inhibitory effects. Seven Eos, namely Aar, CC, CL, ER, FF, GP, and OP, have weak inhibition against DXR (inhibition rates ranging from 10% to 25%). To our delight, five products, such as the oils from ZB, ST, TQ, OV, and EC showed weak to medium inhibitory activity on the enzyme (inhibition rates ranging from 33% to 64%). Further analyses of the five oils indicated that they were all able to suppress the activity of DXR in concentration-dependent manners (data are depicted in Figure 3) and their IC_50_ values were calculated and are listed in Table 1. 

### 2.3. GC and GC-MS Analyses of the Active EOs and DXR Inhibition of the Major Components

To further determine the major components of the five EOs displaying DXR inhibition, GC-MS analyses were carried out and the results, listed in Table S2 in SI (only the compounds with contents above 1% are listed) show that the major components of the EOs were β-myrcene, limonene, carveol, linalool, β-caryophyllene, p-allylanisole, citral, thymol, carvacrol, eugenol, and eugenol acetate (content of around 10% and higher). Subsequently, the 11 main components of the five EOs were further confirmed by GC-FID analyses through co-injections using the commercial compounds as references. Based on the confirmation results, the inhibitory effects of the 11 compounds against DXR were measured and the data show that at a concentration of 20 μg/mL, β-myrcene, limonene, β-caryophyllene, p-allylanisole, citral, and eugenol acetate did not have any detectable inhibition against DXR. Carveol, linalool, and thymol displayed a weak effect on DXR (23.2% ± 2.9%, 22.7% ± 2.8%, and 26.5% ± 1.6%, respectively) and carvacrol and eugenol exhibited medium inhibition against the protein (55.6% ± 4.7% and 68.3% ± 3.9%, respectively).

## 3. Material and Methods

### 3.1. Materials

Fourteen Eos, including AG and CL (Table S1 in SI) were purchased from Ji’an Hairui Natural Plants Company (Jiangxi, Ji’an, China). AD, AA, Aar, FS, MD, NI, PC, and ST were purchased from the Market of Materia Medica in Xian and identified by Prof. Bao-Hua Hao from the College of Life Sciences, Northwest University (Xi’an, China). CLo, EC, FF, IV, ZB, G, Gi, GP, and OP were acquired in the local market. CC and MH were collected in the Botanical Garden of Northwest University. The plasmid pET15b-DXR encoding the *dxr* gene of *E. coli* was a kind gift from Prof. C. D. Poulter from the Chemistry Department of the University of Utah. The bacterial strain *E. coli* BL21 (DE3) is a stock maintained by the institute. The other materials used were as follows: fosmidomycin was from the Toronto Research Chemicals Inc. (North York, ON, Canada); the EO compounds (analytical grade) carvacrol, thymol, limonene, carveol, and eugenol were purchased from Sigma-Aldrich (St. Louis, MO, USA); β-myrcene, β-caryophyllene, linalool, and citral were the products of Alfa Aesar (Tianjin, China); p-allylanisole, and eugenol acetate were from Shanghai Yuanmu (Shanghai, China). The stock solutions of these EO compounds (1.0 mM) were prepared in distilled water containing 1.0% DMSO (*W*/*V*). DXP was synthesized according to procedures previously published by this laboratory [30]. All other chemicals used were of an analytical reagent grade. 

### 3.2. Isolation of Plant EOs

The plant EOs were prepared from 10–50 g air-dried or fresh materials by hydrodistillation for 4 h, using a Clevenger-type apparatus. The amount of individual plant material used depended on whether it was able to generate enough oil for the following tests. Dry roots (e.g., AD, CLo, and NI, see Table S1) and dry fruits (e.g., FC, FF, IV, and ZB) were ground before extraction. Dry flower buds (e.g., EC and MD) were steam distilled directly. Dry and fresh aerial parts (e.g., AA, Aar, MH, PC, and ST), fresh leaves (e.g., CC) and fresh peels (e.g., GP and OP) were cut into small pieces beforehand. Fresh garlic and ginger were pounded and then hydrodistilled. Each oil obtained was then transferred from the apparatus to a 0.5 mL Eppendorf tube containing 10–50 mg magnesium sulfate anhydrous (the quantity of the drying agent used depended on the amount of oil produced). After being dried for 4 h, the oil was centrifuged at 5000 rpm at room temperature for 5 min and the supernatant was transferred to another tube and kept at −20 °C under the protection of nitrogen for later use.

### 3.3. Preparation of Recombinant E. coli DXR

The expression and purification of recombinant *E. coli* DXR were carried out in accordance with the reported procedure [20].

### 3.4. DXR Inhibitory Activity Determination Using Photometric Assay

Assay mixtures comprised of 100 mM Tris-HCl (pH 7.4), 5 mM magnesium chloride, 1.0 mM NADPH, 0.5% (*V*/*V*) DMSO, and 6 μg/mL of DXR in a final volume of 120 μL. Plant essential oil (final conc. 50 μg/mL) or individual EO compound (final conc. 20 μg/mL) was added, and the reaction was started by addition of DXP (final conc. 1.2 mM). In a negative control assay, no oil or individual EO compound was added and in a positive control assay, fosmidomycin (final conc. 1.0 μM) was added instead of EOs or individual EO compound. The mixtures were incubated at 30 °C for 20 min and then boiled in boiling water for 3 min before absorbance at 340 nm was recorded [20]. Subsequently, the DXR inhibitory activities of the EOs and the individual EO compound were calculated using the formula given in SI.

### 3.5. GC and GC-MS Analysis

The EOs showing DXR inhibition (activity >30%) were further examined by GC and GC-MS. GC-FID analyses were performed on a Shimadzu GC2010 gas chromatograph (Kyoto, Japan) coupled with an HP-5 capillary column (30 m × 0.32 mm i.d., film thickness 0.25 μm). The carrier gas was nitrogen of high purity. The injector temperature was 240 °C and the oven temperature was programmed from 55 to 220 °C at a rate of 3 °C/min and finally held for 10 min. For GC/MS analysis, the samples were analyzed using a Shimadzu QP2010 detector in the same column and conditions as those described above. Ion source temperature was 145 °C, energy ionization was 70 eV, EIMS were acquired over the mass range of 35–350 Da, and split was 1/50. The individual component was identified by matching the recorded spectra with the library provided by the instrument software through comparing the base peak and the relative abundance of each peak (Similarity >95%). The main components in the EOs (content around 10% and higher) were further clarified by GC-FID analyses through co-injections of the EOs with the commercial EO compounds. 

## 4. Discussion

As a newly validated effective target for the screening of antimicrobial agents, DXR has attracted much attention and some of its inhibitors, including the clinically useful anti-malaria agents fosmidomycin and its congener FR900098, have recently been elucidated [17]. Current research seeking DXR inhibitors still largely focuses on the synthetic analogues of fosmidomycin and FR900098, and natural sources remain an almost unexplored area [25,26,27,28]. The antimicrobial properties of plant EOs have been recognized for more than half a century [31]. The possible utilization of EOs and/or their components in medical pathology and in the control of plant diseases, as well as in the food industry to control the microorganisms being malgenic for consumers and/or for food spoilage has been pointed out by several studies [1,6,32]. In a previous study, we disclosed that some EO compounds, like thymol, carvacrol, eugenol, geraniol, linaool, and nerol et al., whose antimicrobial activities were determined, show weak to medium DXR inhibition [29]. In order to search for more DXR inhibitors from plant EOs and further explain the antimicrobial mechanism of the oils, we tested the DXR inhibitory activities of 35 plant EOs with proven antimicrobial properties and found that the EOs of ZB, ST, TQ, OV, and EC exhibit weak to medium DXR inhibitory activity. Further analyses pointed out that the major active components in these EOs were carveol, linalool, thymol, carvacrol, and eugenol, respectively.

It has been previously determined that the EOs of TQ, OV, and EC have strong antimicrobial activities and their main active principles are thymol, carvacrol (TQ and OV), and eugenol (EC), respectively [7,12,32]. Our experimental results indicated that the EOs of TQ, OV, and EC possess *E. coli* DXR inhibitory effects and the main reason could be owing to the presence of the major active compounds thymol and carvacrol in TQ and OV and eugenol in EC. Furthermore, the investigations on the antimicrobial mechanism of these EO compounds indicate that they act as antimicrobial reagents because they can damage the integrity of the cell membrane, affecting pH homeostasis and the equilibrium of inorganic ions inside cells [11] or leading to the leakage of cell contents, thus suppressing the growth of the bacteria [13,14,15,16,33]. Investigations also displayed that besides the influence on membrane permeability, eugenol was also able to affect the energy generation of cells through the inhibition of glucose uptake or the utilization of glucose [12], effectively inhibiting the activities of a series of amino acid decarboxylases [34]. Eugenol and carvacrol could efficaciously suppress the activity of ATPase as well [13]. A recent study showed that plant EOs can restrain the growth of Gram-negative bacteria via impairing the mature biofilms generated by this kind of microorganisms [35]. Our observations show that DXR inhibition might be another pathway for these EO compounds to display their antimicrobial abilities. We also found that the DXR inhibition activities of the oils of ST and ZB were much weaker than that of EC and OV oils and comparable to that of TQ oil (Table S1 in SI). This could be because ZB contains only carveol and linalool as its active compounds, which possess weak DXR inhibitory activity, and its other major components like β-myrcene, limonene, and β-caryophyllene possess no DXR activity. Similarly, ST contains linalool and thymol as its active principles, which are both weak DXR inhibitors, and its other main constituents, such as p-allylanisole and citral, have no DXR inhibitory effect. We also obtained some disappointing results; for example, although the strong antimicrobial activity of the EOs of garlic, ginger, and tea tree have been known for years [36,37,38,39], their EOs only exhibit very low (garlic and tea tree, Table S1 in SI) or even undetectable (ginger) DXR inhibitory activities. The data imply that these EOs must exhibit their antimicrobial activities by acting against other targets, such as ATPase, amino acid decarboxylase, and biofilms generated by Gram-negative bacteria. 

## 5. Conclusions

In this study, the DXR inhibitory activities of 35 plant EOs were evaluated and the results showed that the EOs of ZB, ST, TQ, OV, and EC displayed weak to medium inhibition against the target. Further analyses of these five EOs showed that the major active compounds were eugenol, carvacrol, thymol, carveol, and linalool. The research carried out in this study notes that the plant EOs would be a good source for the search of DXR inhibitors. More investigations on plant EOs are necessary to finds more DXR inhibitors that are beneficial to the development of novel antibiotics and herbicides. In addition, the data obtained could also be very helpful for characterizing the antimicrobial modes of action of plant EOs and direct the reasonable use of EOs in the food industry and in the control of phytopathogenic microbials. 

## Figures and Tables

**Table 1:** IC_50_ values of the EOs of ZB, ST, TQ, OV, and EC against DXR.

EOs	IC_50_ (μg/mL)
*Zanbthoxylum bungeanum* (ZB)	78.05 ± 3.37
*Schizonepetae tenuifoliae* (ST)	65.32 ± 2.54
*Thymus quinquecostatus* (TQ)	59.48 ± 3.11
*Origanum vulgare* (OV)	48.37 ± 1.07
*Eugenia caryophyllata* (EC)	37.25 ± 2.58
Fosmidomycin *	0.038 ± 0.014

* Fosmidomycin was used as a positive control at 0.18 μg/mL; the reported IC_50_ for it against *E. coli* DXR is 0.068 μg/mL [25].

## Supplementary Materials

Supplementary materials are available online.
