# Supplementary material for: Inhibitory Activity of Plant Essential Oils against E. coli 1-Deoxy-d-xylulose-5-phosphate reductoisomerase
Source: Molecules. 2019 Jul 10;24(14):2518. doi: 10.3390/molecules24142518 (PMC6681031; doi:10.3390/molecules24142518)
Supplement: Supplementary file 1 [file molecules-24-02518-s001.pdf]

Supporting Information

Inhibitory activity of plant essential oils against *E. coli* 1-deoxy-D-xylulose-5-phosphate reductoisomerase

Ge Yan, Qi Jia, Fang-Lin Tian, Xian Hui, Heng Li, Yi-Ming Li, and Wen-Yun Gao

I. The formula used for the calculation of DXR inhibition of the EOs and the individual EO compound:

$$\% \text{ inhibition of DXR} = \frac{A_{\text{negative control}} - A_{\text{sample}}}{A_{\text{negative control}}} \times 100\%$$

II. Supplementary Tables:

Table S1. Overview of 35 plant EOs and their inhibitory activities against *E. coli* DXR at a concentration of around 0.05 mg/mL.

Plants	Abbr.	Yield of EO (%)	Appearance	Inhibition (%)
Acorus gramineus	AG	CS*	yellowish	4.2 ± 0.4
Angelica dahurica (dry roots)	AD	0.14	yellowish	3.6 ± 0.4
Artemisia annua L. (air-dried aerial parts)	AA	0.37	yellowish	3.8 ± 0.3
Artemisia argyi Levl. (air-dried aerial parts)	AAr	0.18	olive green	12.3 ± 0.9
Cinnamomum camphora (fresh leaves)	CC	0.46	colorless	18.2 ± 1.4
Citrus limonum	CL	CS	yellowish	11.3 ± 1.5
Curcuma longa L. (dry roots)	CLo	6.38	brown	4.2 ± 0.2
Cymbopogon winterrianus	CW	CS	yellowish	3.2 ± 0.5

Eucalyptus robusta	ER	CS	colorless	12.9 ± 0.4
Eugenia caryophyllata (air-dried flower buds)	EC	9.8	yellowish	64.1 ± 3.7
Forsythia suspensa (Thunb.) (air-dried fruits)	FS	2.72	colorless	ND**
Fructus foeniculi (air-dried fruits)	FF	2.23	yellowish	24.3 ± 3.1
Hippophae rhamnoides	HR	CS	sorrelish	2.4 ± 0.5
Illicium verum (air-dried fruits)	IV	3.21	yellowish	2.7 ± 0.6
Jasminum sambac (L.)	JS	CS	yellowish	3.7 ± 0.2
Magnolia denudata Desr. (air-dried flower buds)	MD	2.41	yellowish	ND
Mentha haplocalyx Briq. (fresh aerial parts)	MH	1.74	pistachio	ND
Mentha spicata	MS	CS	colorless	ND
Notopterygium forbesii (air-dried roots/rhizome)	NI	2.34	yellowish	ND
Origanum vulgare L.	OV	CS	yellowish	49.3 ± 5.1
Pogostemon cablin Benth. (air-dried aerial parts)	PC	2.33	sorrelish	3.3 ± 0.4
Rhizome chuanxiong	RC	CS	sorrelish	6.5 ± 0.2
Rosa rugosa	RR	CS	yellowish	ND
Schizonepetae tenuifoliae (air-dried aerial parts)	ST	1.84	yellowish	35.5 ± 3.4
Thymus quinquecostatus C.	TQ	CS	yellowish	39.7 ± 5.7
Zanbthoxylum bungeanum (air-dried fruits)	ZB	1.25	colorless	33.2 ± 3.5
garlic (fresh)	G	0.33	yellowish	6.5 ± 0.4
ginger (fresh)	Gi	0.43	yellowish	ND
grapefruit peels (fresh)	GP	0.54	colorless	14.4 ± 1.4
lavandula	L	CS	yellowish	ND
orange peels (fresh)	OP	0.31	colorless	13.3 ± 0.8
pine	P	CS	colorless	3.1 ± 0.1
sweet almond	SA	CS	yellow	5.3 ± 0.9

tea tree	TT	CS	colorless	4.6 ± 0.2
wintergreen (leaves)	W	CS	colorless	5.2 ± 0.6
fosmidomycin (1.0 μM)				~100

*CS: obtained from commercial sources; **ND: not detectable.

Table S2. GC-MS results of the components (content > 1%) of the five EOs displaying DXR inhibitory activity.

EOs compounds	ZB	ST	TQ	OV	EC
β-myrcene	9.15				
limonene	28.34				
3-octanol				1.94	
carveol	8.62				
camphene	1.24			1.41	
p-cymene			5.61	7.09	
crypton	1.99				
linalool	10.37	9.64	2.43	5.68	
piperitol	2.61				
α-terpineol	5.87				
piperitone	1.61				
β-caryophyllene	7.83	2.32			9.23
α-caryophyllene	1.56	2.52		3.90	2.42
germacrene	2.66				
α-cadinol	2.35				
ledol	2.86				
p-allylanisole		14.35			
cis-citral		15.93			1.13
α-farnesene		5.67			
trans-citral		17.13			

β-farnesene	1.83			
thymol	12.28	22.54	13.53	
1-methyl-3-isopropylbenzene		5.76		
eucalyptol		5.15		
borneol		2.74		
carvacrol		31.49	37.47	
eugenol				71.53
Eugenol acetate				8.20
